# Warts Cured

**Published:** 1885-03

**Authors:** E. W. Berridge

**Affiliations:** London


### CLINICAL BUREAU.

### WARTS CURED.

E. W. Berridge, M. D., London.

*Natrum Muriaticum in Warts.*—February, 1884. A boy about eleven years old had had for three or four years a smooth, hard wart on the ball of left thumb near the first joint. For about six months he had also had one on base of metacarpal bone of left little finger, one on palmar surface of first phalanx of left thumb, and one or two on the base of right palm. He had received a single dose of *Thuja* CM (F.C.) about the end of 1881, which only temporarily reduced the size of the large wart, the only one he then had. A single dose of *Verrucinum* 10M (F.C.) was given in March, 1882, but without result; nor did a repetition of the same nosode in the same potency, twice a day for ten days, nearly five weeks later, produce any better effect. Lastly, in October, 1883, he took a daily dose of *Thuja* CM (F.C.) for some days, but equally without result. Subsequently he took no medicine till the present time.

*Diagnosis of the remedy.*—Lippe's invaluable Repertory gives, "Warts on the palm, *Nat.-mur.*" I accordingly gave him a daily dose of *Nat.-mur.* CM (F.C.).

In five days he showed me that the warts had all gone. I stopped the medicine, and the warts have not returned to this day. He has never been a salt-eater.

Case 9. *Sulphur in Warts.*—Miss M. B., æt. eighteen.

June 23d, 1884.—Has had warts on hands for some months, and on feet since before Christmas, the latter being the oldest. She has now a wart on ulnar side of last phalanx of left second finger; a smaller one on radial side of last phalanx of left forefinger, near the joint; one under nail of left second finger; one under last phalanx of right great toe; and one on sole just behind left little toe. All the warts are similar, no pedicle, hard, rather smooth; always aching, sometimes throbbing, tender to touch.

*Diagnosis of remedy:*—

Warts hard: *Ant.-c., Calc.-dulc., Fluor-ac., Lach., Ran.-bulb., Sil., Sulph.*

Warts painful: *Caust., Nat.-carb., Nat.-mur., Nit.-ac., Sabi., Sulph., Thuja.*

Warts throbbing: *Calc., Caust., Hep., Kali-c., Lyc., Petrol., Sep., Sil., Sulph.*

The other symptoms have not yet been recorded.

I gave her, therefore, *Sulph.* DM (F.C.) (the only remedy that corresponded to the case) twice a day for fourteen days.

July 8th.—Reports that by June 27th all the warts were less painful, the large one on second finger flatter and darker, and that under nail darker. By June 29th the pain had quite gone from all the warts. On July 1st the wart under the nail came completely off. Subsequently the other two warts on the fingers came out, merely leaving holes where they had been. Hardly any mark to show where the wart under the nail had been. Warts on feet remain.

July 23d.—Warts on feet have now gone also; no traces of any left.

October 27th.—No return of warts.

*Comments.*—(1) The first point to be noticed is that it is impossible to treat patients according to pathological theories—that is, according to the name of the disease. The routine pathological practitioner would argue that warts were warts, and that the treatment of each must be the same. But we see here that different remedies are indicated in the different cases.

(2) The failure of *Verrucinum* in the first case also demonstrates the fallacy of Lux's system of Isopathy. Yet *Verrucinum* has, in the hands of a colleague, cured a very bad case. No nosode will cure every case of the corresponding disease; to prescribe it thus is to commit a "fatal error." It will only cure when homœopathic, not merely to the objective symptom, sometimes called by pathological prescribers the "disease," but also to the totality of the symptoms of the individual patient, whether we can trace the connection between all these symptoms and the "disease" or not.

(3) In the first case, the locality of the warts was the keynote in the selection of the remedy, the other symptoms being vague; in the second case, the locality being less defined, and the subjective symptoms marked, the latter become the keynote. This shows how, in different cases, different elements may have priority in value; in one case the locality may be the keynote, in another the character of the pain, in another the conditions, or in another the concomitants. But keynotes should never be relied upon exclusively, but only as leading to the remedy which corresponds to the totality of the symptoms.

(4) It will be noticed that the first case was cured quicker than the second, though it had been of much longer duration. This is accounted for by the fact that in the first case the unfavorable surroundings of worry, past allopathic treatment, etc., did not exist as they did in the second.

(5) In the second case the warts disappeared in the inverse order of their appearance. This is another confirmation of Hahnemann's teaching, and is the great criterion of a *permanent* cure. In the first case the order of disappearance was unfortunately not observed.

(6) For future verification, it may be noticed that the warts cured by *Nat.-mur.* went from left to right; those cured by *Sulphur*, from below upward.—*Homœopathic World.*

